# Application of shear wave elastography in the assessment of renal cortical elasticity in patients with hypertension

**DOI:** 10.3389/fmed.2025.1624558

**Published:** 2025-09-26

**Authors:** Miaolei Dai, Liang Wang, Jianfeng Luo

**Affiliations:** ^1^Department of Ultrasound, Integrated Traditional Chinese and Western Medicine Hospital of Wenzhou Medical University, Wenzhou, China; ^2^Department of Ultrasound, The Second Affiliated Hospital and Yuying Children’s Hospital of Wenzhou Medical University, Wenzhou, China; ^3^Wenzhou Key Laboratory of Structural and Functional Imaging, Wenzhou, China

**Keywords:** ultrasound, shear wave, elasticity, hypertension, renal cortex

## Abstract

**Objective:**

To evaluate the clinical utility of shear wave elastography (SWE) for measuring renal cortical elasticity in patients with hypertension.

**Methods:**

Following the diagnostic criteria established in the 2020 International Society of Hypertension Global Hypertension Practice Guidelines, 44 patients with essential hypertension from our hospital’s Department of Cardiology were enrolled alongside 46 healthy controls during the same period. General demographic data and blood biochemical indicators of renal function were documented for all participants. All subjects underwent two-dimensional ultrasound and SWE examinations to obtain conventional ultrasound parameters and Young’s modulus (YM) values of the right renal cortex. Parameters were compared between the two groups. Multiple linear regression analysis was conducted to identify relationships between the YM value of the right renal cortex and general demographic data, conventional ultrasound indicators, and renal function parameters in the hypertensive group.

**Results:**

Disease duration, systolic blood pressure, and diastolic blood pressure in the hypertensive group were significantly higher than those in the control group (*p* < 0.05). The YM value of the right renal cortex in the hypertensive group was significantly higher than that in the control group (*p* < 0.05). Correlation analysis revealed a strong positive correlation between the YM value of the right renal cortex and the duration of hypertension (*r* = 0.747, *p* < 0.001). No significant correlations were found between right renal cortex YM and age, blood pressure, right renal volume, right renal cortex thickness, peak systolic velocity, resistive index of the right renal artery trunk, blood urea nitrogen, blood creatinine, or uric acid (*p* > 0.05). Multivariate linear regression analysis identified duration of hypertension as an independent predictor of right renal cortex YM. The regression equation was determined to be Y = 8.63 + 0.41 X_1_.

**Conclusion:**

SWE may serve as a valuable non-invasive screening tool for early detection of hypertension-related renal changes before conventional parameters show abnormalities. The positive correlation between cortical stiffness and hypertension duration suggests SWE could assist clinicians in risk stratification and monitoring progression of subclinical renal damage, potentially enabling earlier therapeutic interventions to prevent progression to chronic kidney disease.

## 1 Introduction

Hypertension affects over 1 billion individuals worldwide (1, 2), with approximately 20–40% of patients ultimately developing chronic kidney disease (CKD) (3), and some progressing to end-stage renal disease requiring dialysis or transplantation (4). Early diagnosis and intervention for hypertension-related renal impairment remains a critical challenge (5). Conventional parameters such as serum creatinine and blood urea nitrogen often fail to reflect subtle structural alterations in early stages due to robust renal compensatory capacity, posing significant challenges for early diagnosis of hypertension-related nephropathy. The renal cortex, where functional nephrons are concentrated, is initially affected by pathological processes including renal arteriolar sclerosis and interstitial fibrosis secondary to hypertension (6). Therefore, precise assessment of alterations in renal cortical elasticity may provide critical information for early detection and monitoring of hypertension-related renal injury (7).

Shear Wave Elastography (SWE) has gained widespread application in hepatic fibrosis assessment, thyroid nodule differentiation, and breast lesion evaluation due to its non-invasive, quantitative, and real-time capabilities (8, 9). SWE reflects tissue stiffness by measuring shear wave propagation velocity within tissues. Compared to conventional ultrasonography, which primarily evaluates renal morphology and echogenic characteristics, SWE demonstrates superior sensitivity in detecting renal parenchymal elasticity and identifying pathological changes such as renal fibrosis and tissue remodeling (10). Previous literature has documented that SWE demonstrates excellent discriminatory capacity across different CKD stages, with elasticity parameters correlating with glomerular filtration rate (eGFR) and serum creatinine levels (11). However, research regarding SWE assessment of renal elasticity in hypertensive patients remains relatively limited, with insufficient standardized technical parameters and operational protocols. Additionally, multiple potential factors influence renal cortical elasticity, including patient age, blood pressure control status, and renal arterial perfusion, with their independent mechanisms of action not fully elucidated.

Although alterations in renal elasticity are intimately associated with tissue remodeling, whether specific elasticity value changes possess independent clinical diagnostic significance requires more comprehensive research support (12). The current clinical application of SWE in assessing hypertension-related renal damage remains exploratory, with considerable uncertainty regarding severity determination and progression trend through elasticity parameters (13). Therefore, integration of patient demographic information, laboratory parameters, and imaging data is essential for comprehensive analysis of clinical factors influencing renal cortical elasticity, establishing the foundation for precise SWE application in hypertensive patients.

This study aims to employ SWE technology for quantitative analysis of renal cortical elasticity in hypertensive patients and investigate its correlation with clinical characteristics and laboratory parameters, thereby preliminarily identifying independent clinical factors potentially influencing renal cortical elasticity. Through this investigation, we intend to provide preliminary data supporting the establishment of SWE-based assessment criteria for hypertension-related renal damage in future applications, while offering novel perspectives and methodologies for early intervention and personalized therapeutic approaches.

## 2 Methods

### 2.1 General information

This investigation was conducted in accordance with the Declaration of Helsinki and received approval from the Institutional Ethics Committee.

A prospective cohort of 44 patients diagnosed with essential hypertension was recruited from the Department of Cardiology between November 2021 and August 2022. The study population comprised 21 males and 23 females, with an age range of 29–73 years. Inclusion criteria were established as follows: ① Confirmed hypertension diagnosis according to the 2020 International Society of Hypertension guidelines (14), defined as repeated measurements of systolic blood pressure >140 mmHg and/or diastolic blood pressure >90 mmHg, documented through 2–3 measurements over a 1–4 week period, ② Suboptimal blood pressure control within the preceding 3 months, defined as: For patients <65 years: blood pressure ≥130/80 mmHg, For patients ≥65 years: blood pressure ≥140/90 mmHg, ③ Normal biochemical parameters, urinalysis, renal function indices, and conventional ultrasonographic findings, ④ Current use of antihypertensive medications with documented suboptimal blood pressure control. Exclusion criteria encompassed: ① Primary and secondary glomerular pathologies (including glomerulonephritis and diabetic nephropathy), ② Space-occupying renal lesions, ③ Urinary tract infections, ④ Congenital urinary tract malformations.

A control group of 46 age- and sex-matched healthy volunteers (22 males, 24 females; age range 25–74 years) was recruited concurrently. Control subjects met the following criteria: ① No history of hepatic, renal, or other chronic conditions affecting renal function, ② Normal biochemical profiles, ③ Unremarkable urinalysis results, ④ Normal conventional ultrasonographic findings, ⑤ No current use of medications known to affect renal function or hemodynamics.

### 2.2 Data collection and laboratory parameters

Demographic and clinical data were systematically collected for all participants, including gender, age, systolic blood pressure, diastolic blood pressure, and duration of hypertension. Laboratory parameters obtained at admission: blood urea nitrogen, blood creatinine, and uric acid.

### 2.3 Instruments and equipment

Imaging Equipment Ultrasonographic examinations were performed using a Mindray Resona7 color Doppler ultrasound system (Mindray Medical International Limited, Shenzhen, China). The Mindray Resona7 is equipped with SC5-1 U convex array transducer (frequency range: 1–6 MHz) and Sound Touch Quantification (STQ) shear wave elastography module.

### 2.4 Ultrasonographic protocol

All examinations were performed by two ultrasound specialists, each with more than 5 years of specialized experience in ultrasound elastography, using standardized protocols and identical equipment. The subject is examined in a prone position. First, the length, width, thickness, cortical thickness, peak systolic velocity (PSV), end diastolic velocity (EDV), and resistive index (RI) of the right kidney are measured on a two-dimensional ultrasound image. Hemodynamic parameters such as the right kidney volume are calculated using the following formula: kidney volume = length diameter × width diameter × thickness diameter × *π*/6; Then take the long axis section of the kidney perpendicular to the sound beam, activate the STQ mode, and instruct the subject to hold their breath until the image stabilizes (respiratory motion index ≥ 4 stars). Select the renal cortex area under the renal capsule in the middle of the right kidney as the area of interest, avoiding the renal sinus. Repeat the detection 6 times in each part of the renal cortex, calculate the average value, and obtain the YM value of the right renal cortex. This measurement protocol was followed consistently by both operators to ensure standardized data acquisition.

### 2.5 Statistical methods

SPSS 26.0 statistical software is used for data analysis. Metric data that follow a normal distribution are expressed as mean ± standard deviation ( $\bar{x}$ ± s). The *t*-test is used to compare data between two groups that follow a normal distribution and have the same variance. Pearson correlation analysis was used for the correlation analysis between variables. For variables that are meaningful for univariate analysis, a simple linear regression model is used to analyze the key factors of YM values in the right renal cortex. *p* < 0.05 indicates a statistically significant difference.

## 3 Results

### 3.1 Baseline characteristics

There was a statistically significant difference (*p* < 0.05) in the duration of hypertension, systolic blood pressure, and diastolic blood pressure between the healthy control group and the simple hypertension group. There were no statistically significant differences in age, gender ratio, right kidney volume, right renal cortex thickness, PSV and RI of the right renal artery trunk, blood urea nitrogen, blood creatinine, and uric acid between the two groups (*p* > 0.05) (Table 1).

**Table 1:** Comparison of general information, conventional ultrasound parameters, and renal function laboratory indicators between healthy control group and essential hypertension group ( $\bar{x}$ ± s).

Parameters	Healthy control group	Essential hypertension group	*t*	*p*
Age (years)	55.91 ± 12.87	54.38 ± 15.82	0.50	0.62
Gender (Male/Female)	22/24	21/23	–	–
Systolic blood pressure (mmHg)	106.61 ± 12.18	136.48 ± 17.21	9.46	0.00
Diastolic blood pressure (mmHg)	66.39 ± 10.37	81.39 ± 14.05	5.74	0.00
Duration of hypertension (months)	0.00	18.20 ± 6.66	18.12	0.00
Right kidney volume (ml)	107.91 ± 21.59	116.07 ± 40.40	1.19	0.24
Right renal cortical thickness (mm)	7.56 ± 1.07	7.89 ± 1.34	0.30	0.31
Right renal artery main trunk PSV (m/s)	0.80 ± 0.24	0.77 ± 0.15	−0.91	0.37
Right renal artery main trunk RI	0.64 ± 0.07	0.66 ± 0.08	1.37	0.17
Blood urea nitrogen (mmol/L)	4.86 ± 1.33	4.93 ± 1.02	0.27	0.79
Serum creatinine (μmol/L)	44.48 ± 13.36	45.63 ± 12.58	0.42	0.68
Uric acid (μmol/L)	299.59 ± 63.44	310.00 ± 66.53	0.76	0.45

### 3.2 SWE measurements

**Figure 1:**
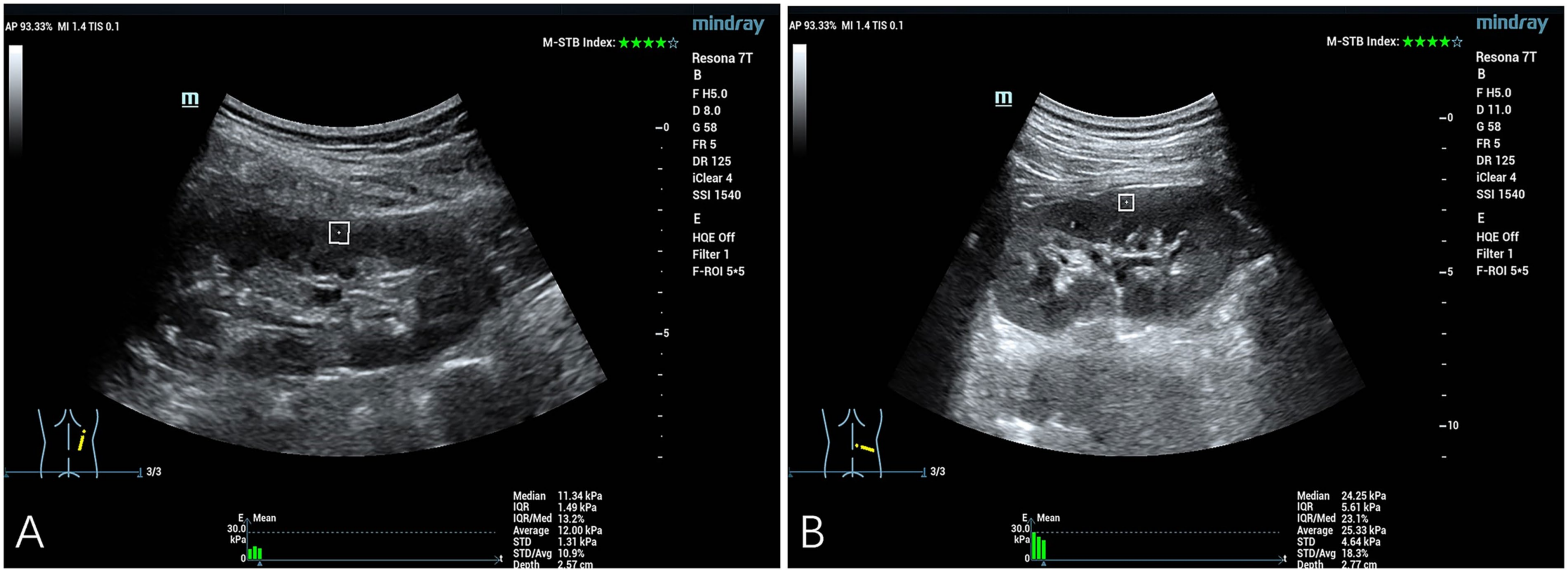
Quantitative assessment of SWE in the right renal cortex. **(A)** The YM measurement obtained from the right renal cortex of a representative healthy control subject was 11.34 kPa. **(B)** The YM measurement obtained from the right renal cortical tissue of a patient diagnosed with hypertension demonstrated an elevated value of 24.25 kPa.

Comparison of YM values in the right renal cortex between the control group and the simple hypertension group: The YM value in the right renal cortex of the simple hypertension group was higher than that of the control group, and the difference was statistically significant (*p* < 0.05) (Table 2 and Figure 1).

**Table 2:** Comparison of right renal cortical YM values between healthy control group and hypertension group ( $\bar{x}$ ± s).

Parameter	Healthy control group	Essential hypertension group	*t*	*p*
Right renal cortex YM value (kPa)	13.38 ± 2.55	16.10 ± 3.66	4.07	0.00

### 3.3 Correlation analysis and linear regression analysis

Correlation analysis between right renal cortex YM value and general information, routine ultrasound indicators, and renal function indicators in the simple hypertension group, and simple linear regression analysis showed a strong positive correlation between right renal cortex YM value and hypertension course (*r* = 0.747, *p* < 0.001). The YM value of the right renal cortex was not correlated with age, blood pressure, right renal volume, right renal cortex thickness, right renal artery trunk PSV, right renal artery trunk RI, blood urea nitrogen, blood creatinine, and uric acid (*p* > 0.05) (Table 3). Further simple linear regression analysis was conducted with the YM value of the right renal cortex as the dependent variable (Y) and the duration of hypertension (X_1_) as the independent variable. The results showed that the duration of hypertension was an independent influencing factor of right renal cortex YM, and the regression equation was Y = 8.63 + 0.41 X_1_(Table 4).

**Table 3:** Correlation analysis between right renal cortical YM value and general data, conventional ultrasound parameters, and renal function laboratory indicators in hypertension group.

Parameters	Right renal cortical YM value	
	*r*	*P*
Age (years)	−0.187	0.225
Systolic blood pressure (mmHg)	0.076	0.626
Diastolic blood pressure (mmHg)	−0.014	0.928
Duration of hypertension (months)	0.747*	0.000
Right kidney volume (ml)	−0.041	0.790
Right renal cortical thickness (mm)	0.000	0.999
Right renal artery main trunk PSV (m/s)	0.109	0.482
Right renal artery main trunk RI	−0.192	0.211
Blood urea nitrogen (mmol/L)	−0.163	0.291
Serum creatinine (μmol/L)	0.163	0.290
Uric acid (μmol/L)	0.019	0.902

**Table 4:** Simple linear regression analysis of right renal cortical YM value in hypertension group.

Indicator	Regression coefficient	Standard error	Standardized regression coefficient	*t*	*p*
Constant	8.63	1.09		7.92	0.00
Duration of hypertension	0.41	0.06	0.75	7.29	0.00

## 4 Discussion

Hypertension is a common and frequently occurring disease in clinical practice, and long-term hypertension often affects the kidneys and eventually develops into hypertensive nephropathy (15). With the increasing incidence rate and mortality of hypertensive nephropathy, clinical attention to hypertensive nephropathy is gradually increasing (16). Due to the delayed and non-specific results of laboratory and imaging examinations, how to detect and intervene in the treatment of hypertensive nephropathy as early as possible is currently a major challenge in the medical field (17). Although renal biopsy is still the “gold standard” for this disease, in practical work, the proportion of hypertensive patients diagnosed with kidney damage through clinical laboratory examination and imaging examination is higher than that diagnosed by renal biopsy pathology (18). Due to the partial similarity in pathological characteristics between hypertensive nephropathy and various primary or secondary kidney diseases, this method also has certain limitations (19).

Conventional ultrasonography has established its role in hypertension prognosis, particularly through Doppler assessment of renal hemodynamics. The renal RI has been extensively validated as a marker of vascular damage in cardiovascular diseases and predictor of adverse outcomes in hypertensive patients (20, 21). In this study, there was no statistically significant difference in right renal artery trunk PSV and RI between the two groups (*p* > 0.05). Due to the fact that the selected case group in this study were all patients with simple hypertension, patients were only found to have elevated blood pressure during blood pressure measurement. These patients presented without characteristic hypertensive symptoms, and conventional ultrasound examination revealed no significant alterations in renal hemodynamics. Although our study did not demonstrate significant RI differences between groups, this finding is consistent with existing literature suggesting that RI changes may become apparent primarily in advanced stages of hypertensive nephropathy. Recent investigations have explored alternative Doppler parameters, such as the renal augmented velocity index, which may demonstrate superior sensitivity for detecting early vascular changes compared to traditional RI measurements in hypertensive populations (22).

SWE represents an emerging ultrasonic technique that has garnered significant attention in renal disease evaluation. SWE can detect the hardness of the renal cortex and reflect its biomechanical changes (23). In our study, the YM in the right renal cortex of the simple hypertension group was higher than that of the healthy control group, indicating that hypertension may lead to an increase in renal cortex hardness (24). In hypertensive patients, although no significant changes were found in renal anatomy, hemodynamics, and physiological function, the cortical hardness of the kidneys has already changed, which is consistent with previous research results (25). This may be because when patients have long-term high blood pressure, it can lead to damage to renal vascular endothelial cells. On the one hand, the walls of renal arterioles thicken, and on the other hand, the renin angiotensin aldosterone system is activated. The glomeruli have mild hypoxic ischemic pathological changes, and occasionally segmental glomerular fibrosis (26, 27). At this time, renal damage caused by hypertension is mainly manifested as benign glomerular arteriosclerosis. Although focal glomerular fibrosis has occurred, the kidneys can maintain relatively constant renal blood flow and renal capillary hydrostatic pressure through a series of compensatory adjustments, so there will be no changes in renal structure and function due to blood pressure fluctuations in the short term. If patients do not receive timely and effective intervention at this time, the severity of kidney involvement will progressively worsen and eventually develop into uremia (28, 29). Therefore, this research result has significant clinical implications.

In this study, the strong positive correlation between hypertension duration and cortical stiffness, with hypertension duration identified as an independent predictor of renal cortical Young’s modulus, indicates that SWE may serve as a valuable biomarker for monitoring subclinical disease progression. Regarding the underlying pathophysiology, hypertension-induced renal damage represents a progressive pathological process. Early hypertensive nephropathy manifests as benign arteriolosclerosis (30, 31), characterized by renal arterial intimal thickening, subsequent vessel wall hypertrophy with luminal narrowing, mild glomerular ischemic changes, and focal glomerular and tubular atrophy. This progression perpetuates a pathological cycle wherein renal damage exacerbates hypertension severity, which subsequently accelerates further renal deterioration. In the late stage, malignant glomerular arteriosclerosis develops, characterized by glomerular consolidation and sclerosis, renal interstitial fibrosis, arteriolar fibrinoid necrosis or thrombosis, and associated pathological changes (32). Concurrently, renal tissue stiffness progressively increases throughout this pathological continuum. Multiple investigations have demonstrated the clinical utility of elastography in various renal pathologies, establishing its role as a non-invasive assessment tool for tissue mechanical properties. Leong et al. validated SWE’s capability to quantify renal tissue stiffness, demonstrating superior performance compared to conventional ultrasound parameters in detecting CKD (33). Chen et al. demonstrated significant correlations between renal stiffness measured by SWE and histopathological renal fibrosis, suggesting elastography’s utility as a surrogate marker for early fibrosis detection (34). Similarly, investigation by Leong et al. found that SWE accurately detects chronic renal histopathological changes, including glomerular sclerosis, interstitial fibrosis, and tubular atrophy (35). These findings support our observation that SWE can detect subclinical renal changes before conventional parameters become abnormal.

The absence of significant correlations between renal cortical Young’s modulus and several conventional renal parameters in our study warrants detailed discussion. The lack of correlation with renal volume measurements suggest that morphological changes may lag behind biomechanical alterations in early renal disease. The non-significant relationship between cortical stiffness and conventional Doppler parameters (PSV and RI) in our cohort reflects the limitations of hemodynamic assessments in detecting early renal damage. This finding is consistent with study demonstrating that RI measurements remain within normal ranges during initial stages of renal pathology, only becoming elevated with advanced vascular changes (36). Our results suggest that elastographic measurements may provide superior sensitivity for detecting early pathological changes compared to conventional hemodynamic parameters. Additionally, the absence of correlation between cortical Young’s modulus and serum biomarkers (blood urea nitrogen, creatinine, and uric acid) in our study population reflects the preserved renal function in early hypertensive disease. This observation is particularly significant, as it demonstrates SWE’s ability to detect subclinical renal alterations before conventional functional markers become abnormal. Limitations of this study: (1) The single-center design and limited sample size may restrict generalizability and statistical power, necessitating larger multi-center studies for validation; (2) There is a lack of comparison between hypertensive nephropathy groups in this study; (3) The study population comprised exclusively early-stage hypertensive patients with suboptimal pharmaceutical control, without comparison to populations achieving target blood pressure ranges following therapeutic intervention; (4) The involvement of two different ultrasound operators, despite their comparable expertise in elastography techniques, introduces potential inter-operator variability that may influence measurement consistency; (5) We did not systematically analyze the potential effects of antihypertensive medications on renal stiffness measurements or evaluate the impact of blood pressure variability on elastographic findings; (6) This study lacks direct biochemical assessment of RAS activation markers, such as urinary angiotensinogen, which would provide mechanistic validation for the observed correlation between hypertension duration and renal cortical stiffness. These limitations should be considered when interpreting our findings and designing future investigations in this field

## 5 Conclusion

In summary, for patients with simple hypertension, although routine clinical screening has not yet found any damage to renal structure and physiological function, the hardness of the right renal cortex has changed. And the duration of hypertension is an independent influencing factor. Clinically, SWE could be integrated into routine hypertensive patient evaluation as an early screening tool, with hypertension duration serving as a key consideration for patient selection given its role as an independent predictor of renal cortical stiffness, enabling risk stratification, personalized monitoring, and earlier therapeutic interventions when conventional biomarkers remain normal. The technique’s ability to prompt more aggressive blood pressure management, earlier nephrology consultation, and enhanced monitoring protocols, combined with its seamless incorporation into existing ultrasound examinations with minimal additional cost and time burden, positions elastography as a valuable auxiliary diagnostic tool that represents a paradigm shift toward earlier, more sensitive detection of hypertensive renal involvement, ultimately offering clinicians an evidence-based approach for optimizing patient care through timely intervention and personalized management strategies.

## Data Availability

The raw data supporting the conclusions of this article will be made available by the authors, without undue reservation.
